# Leveraging epidemiology as a decision support tool during the COVID-19 epidemic in South Africa

**DOI:** 10.7196/SAMJ.2022.v112i5b.16061

**Published:** 2022-05-31

**Authors:** S P Silal, M J Groome, N Govender, J R C Pulliam, O P Ramadan, A Puren, W Jassat, E Leonard, H Moultrie, K G Meyer-Rath, W Ramkrishna, T Langa, T Furumele, D Moonasar, C Cohen, S Walaza

**Affiliations:** 1 Modelling and Simulation Hub, Africa (MASHA), Department of Statistical Sciences, University of Cape Town, South Africa; 2 Nuffield Department of Medicine, Centre for Global Health and Tropical Medicine, Oxford University, UK; 3 Division of Public Health Surveillance and Response, National Institute for Communicable Diseases, Johannesburg, South Africa; 4 School of Pathology, Faculty of Health Sciences, University of the Witwatersrand, Johannesburg, South Africa; 5 South African DSI-NRF Centre of Excellence in Epidemiological Modelling and Analysis (SACEMA), Stellenbosch University, South Africa; 6 World Health Organization, Emergency Preparedness and Response (EPR), Pretoria; 7 Centre for HIV, National Institute for Communicable Diseases, Johannesburg, South Africa; 8 Division of Virology, School of Pathology, University of the Witwatersrand Medical School, Johannesburg, South Africa; 9 Clinton Health Access Initiative, COVID-19 Programme, Pretoria, South Africa; 10 Centre for Tuberculosis, National Institute for Communicable Diseases, Johannesburg, South Africa; 11 Health Economics and Epidemiology Research Office, University of the Witwatersrand, Johannesburg, South Africa; 12 School of Public Health, Boston University, Boston, USA; 13 National Department of Health, Communicable Diseases, Johannesburg, South Africa; 14 Centre for Respiratory Disease and Meningitis, National Institute for Communicable Diseases, Johannesburg, South Africa; 15 School of Public Health, Faculty of Health Sciences, University of the Witwatersrand, Johannesburg, South Africa

## Abstract

By May 2021, South Africa (SA) had experienced two ‘waves’ of COVID-19 infections, with an initial peak of infections reached in July 2020, followed by a larger peak of infections in January 2021. Public health decisions rely on accurate and timely disease surveillance and epidemiological analyses, and accessibility of data at all levels of government is critical to inform stakeholders to respond effectively. In this paper, we describe the adaptation, development and operation of epidemiological surveillance and modelling systems in SA in response to the COVID-19 epidemic, including data systems for monitoring laboratory-confirmed COVID-19 cases, hospitalisations, mortality and recoveries at a national and provincial level, and how these systems were used to inform modelling projections and public health decisions. Detailed descriptions on the characteristics and completeness of individual datasets are not provided in this paper. Rapid development of robust data systems was necessary to support the response to the SA COVID-19 epidemic. These systems produced data streams that were used in decision-making at all levels of government. While much progress was made in producing epidemiological data, challenges remain to be overcome to address gaps to better prepare for future waves of COVID-19 and other health emergencies.

SARS-CoV-2, a novel coronavirus, was first detected in Wuhan, China, in December 2019 and rapidly spread globally, culminating in characterisation of the coronavirus disease 2019 (COVID-19) as a pandemic by the World Health Organization (WHO) on 11 March 2020.^[1]^ By the end of March 2021 over 126 million confirmed COVID-19 cases and over 2.75 million deaths attributed to COVID-19 had been detected worldwide.^[2]^ South Africa (SA) reported its first imported COVID-19 case on 5 March 2020 with subsequent spread into the community. By May 2021, the country had experienced two ‘waves’ of COVID-19 infections, with an initial peak of infections reached in July 2020, followed by a far larger peak of infections in January 2021 (Fig. 1).^[3]^

Epidemiological surveillance has always played a critical role in the understanding of the distribution and determinants of health in SA, and the COVID-19 epidemic is no exception. Public health decisions rely on accurate, timely disease surveillance and epidemiological analyses. Integration and accessibility of data at national, provincial and district levels are critical to inform all relevant stakeholders, and to guide policies to control transmission of SARS-CoV-2 and guide resource allocation.^[4]^

In this paper, we describe and reflect on the rapid development and implementation of epidemiological surveillance and modelling systems in SA in response to the COVID-19 pandemic, including data systems for monitoring laboratory-confirmed COVID-19 cases, hospitalisations, mortality and recoveries at national and provincial levels, and how these systems were used to inform modelling projections and public health decisions during the first two waves of COVID-19 until March 2021.

## Data system development

Data systems for reporting cases, hospital admissions, deaths and their onward use in modelling projections for COVID-19 in SA are described.

### Cases

Case definitions for confirmed and suspected cases were developed and adapted as the SARS-CoV-2 virus detection expanded from travellers entering SA to widespread community transmission. COVID-19 was defined as a notifiable medical condition necessitating immediate notification to local health authorities. Initially the National Institute for Communicable Diseases (NICD) served as the referral laboratory for COVID-19 diagnostic testing and a laboratory surveillance database was created to capture COVID-19 test and case data in the Research Electronic Data Capture (REDCap) repository and facilitate notification.

As case numbers escalated rapidly during the first wave, the limited capability and restricted access of this database resulted in data quality challenges and delays in case reporting. As private sector laboratories and the National Health Laboratory Services (NHLS) network of laboratories developed capacity for SARS-CoV-2 testing amid growing public and clinical demand, collation of test results was required for national reporting. In response, the NICD developed a COVID-19 case database using XML Web Services called the Notifiable Medical Conditions Surveillance System (NMCSS) line list. The national case line list was updated daily using data extracted from the NHLS data warehouse and data transmitted by all private laboratories. Data were de-duplicated, and cases re-allocated based on provincial inputs. This line list was used for epidemiological analysis, daily and weekly situational reports to all stakeholders (Table 1), and provincial line lists were distributed to departments of health in each of the nine provinces daily to enable provincial response activities. In addition, the web-based database was made accessible to authorised users from any web-enabled electronic device through a secure request portal. Over time the NMCSS has evolved and improved, utilising ArcGIS geocoding to enrich district-, subdistrict-, and ward-level information for cases. It supports a number of informative dashboards generated from the available data including the South African COVID-19 Modelling Consortium (SACMC)’s district-level resurgence monitor, the SACMC Epidemic Explorer.

### Hospitalisation and in-hospital deaths

The NICD implemented Data for COVID (DATCOV), a hospital surveillance system, in late March 2020. DATCOV utilised the WHO clinical case report form to develop an online platform for data entry that allowed public and private sector hospitals to submit data on hospital admissions for patients diagnosed with COVID-19 and provided real-time analysis and reporting.

The first public hospitals to report on DATCOV were the few central and tertiary hospitals in each province designated as COVID-19 treatment facilities in the early stages of the epidemic. By 15 July 2020, the National Department of Health (NDoH) adopted DATCOV as a national hospital surveillance system for COVID-19 and provided support and resources to roll out to all public hospitals. By October 2020, all hospitals in SA with COVID-19 cases were reporting to DATCOV. As new hospitals were enrolled, all admissions since the start of the epidemic were captured retrospectively. The NDoH placed data capturers at each hospital in seven provinces, to facilitate DATCOV data completeness and quality. By 31 March 2021, 250 455 COVID-19 admissions and 51 527 in-hospital deaths were reported from 644 facilities (393 public sector and 251 private sector) throughout the country.

### Recoveries and deaths reported by the provinces

Owing to independent data systems and structures, while the national systems were being established to track epidemiological trends, provinces developed their own methods of data collection in parallel. The systems used to report on COVID-19 deaths and recoveries varied between provinces, with the majority based on Microsoft Excel spreadsheets, which provinces compiled and submitted to a central person at the NDoH for collation and publication. Both in- and out-of-hospital deaths were reported.

Attempts were made to improve COVID-19 death reporting based on the WHO international guidelines,^[5,6]^ though challenges still persist, with substantial delays in the reporting of deaths after they occur. DATCOV has since developed a community deaths module which will generate the mortality line list for provinces. This will assist with mortality data being reported more timeously.

Provinces interpreted the definition of a COVID-19 recovery^[7]^ differently, with some phoning each COVID-19 case to establish recovery, while others used an algorithm based on cases to determine how many people would have recovered. Challenges also arose for reporting of deaths, recoveries and re-infections when patients were diagnosed/treated in districts/provinces outside of their usual residence/place of diagnosis. While recoveries continued to be reported, its usefulness as an indicator has been questioned and is under review.

### Modelling and SACMC Epidemic Explorer

The SACMC was formed in March 2020 as a collaborative effort between academic and government structures to develop COVID-19 model-based projections. To date, the SACMC has produced a series of internal and public-facing reports providing scenario-based projections based on the National COVID Epi Model (NCEM),^[8]^ a dynamic transmission model. The initial model structure and parameters were developed based on international understanding and estimates, combined with local expert opinion, with subsequent updates being refined to incorporate SA-specific data from the NMCSS, DATCOV and other studies to reflect local realities. The NCEM continues to be used to project the number of infections, detected cases, hospitalisations (general ward and intensive care unit), and deaths over time under different transmission scenarios.

**Fig. 2:**
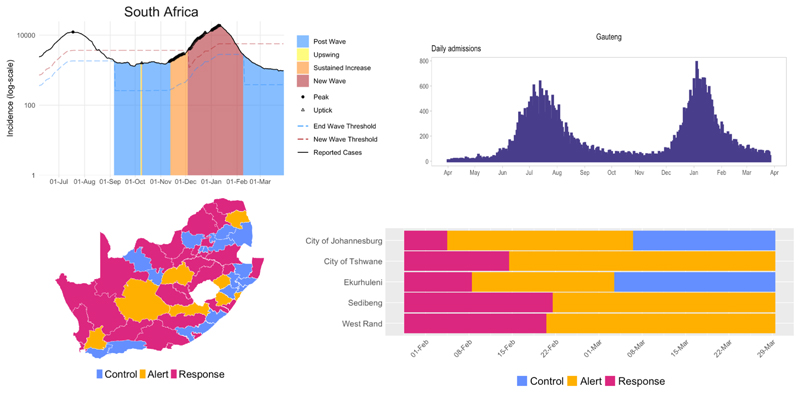
Resurgence monitoring of cases and hospital admissions displayed on the SACMC Epidemic Explorer.^[9]^

In addition to producing model-based projections, the SACMC developed tools and metrics to monitor the status of the epidemic at various administrative levels (national through subdistrict). The SACMC’s Epidemic Explorer dashboard (Fig. 2) displays data and metrics calculated from the DATCOV database and NMCSS’s confirmed case line list, which allow tracking of trends and classify districts into the ‘control’, ‘alert’, and ‘response’ categories outlined in the Incident Management Team (IMT)’s resurgence plan.^[9]^

## Discussion

The decision-making pipeline is complex and the pathway from data to policy to implementation requires inputs from several domains and stakeholders. The data provided by the systems described in this paper contributed only one aspect of these inputs. Attributing policy change to the existence of the systems is not always possible, but the data provided by these systems, and the channels of communication established in response, enabled timely and consistent input into the decision-making pipeline.

Once the first COVID-19 case had been identified, epidemiological indicators were tracked on a daily basis. These indicators include those described in the findings, new cases, testing rate, percentage testing positive, active cases, hospital admissions, and mortality. These data were monitored continuously and analysed to inform interventions. When indicators pointed to a surge in COVID-19 cases in a particular geographical location, a series of responsive actions would be set in motion and the health system’s ability to cope would be assessed and capacity strengthened where necessary, as outlined in the National Intra-Action Review^[10]^ and in the plan of action to mitigate a COVID-19 resurgence in SA (internal NDoH document).

The data from the NMCSS were successfully used to inform the national COVID-19 situation and a number of research, surveillance and modelling initiatives. The surveillance data have been used to describe the epidemiological trends and timing of the two waves of COVID-19 infections experienced and how these varied by province and age groups. The data from the paediatric reports provided useful information during discussions on school closures; these reports highlighted that children were less affected by COVID-19 and constituted <10% of laboratory-confirmed COVID-19 cases in SA.

In addition to monitoring hospital utilisation, the DATCOV data were analysed to demonstrate an increased risk of in-hospital mortality associated with HIV and tuberculosis (TB), as well as other described risk factors for COVID-19. DATCOV also provided useful analysis of the trend in mortality between the first and second wave. DATCOV established the first longitudinal cohort of patients in SA followed up post hospitalisation to determine the prevalence of and risk factors for ‘long COVID’.

The SACMC’s projections have been used to support planning processes across national and provincial government, including for facility readiness (number of hospital beds, staff, drugs, ventilators, and field hospitals needed), laboratory services (number of tests needed and testing algorithms), environmental health (number and placement of mortuary containers), and the national and provincial treasuries. Output from the Epidemic Explorer is displayed at the weekly IMT meetings and frequently accessed by members of the Ministerial Advisory Committee on COVID-19, NICD staff and provincial health departments, with an open-access version of the dashboard made available to the public.

Both modelling and epidemiological data were used to determine the type and quantity of personal protective equipment (PPE) that was required at various stages of the pandemic. The PPE forecasts were also used to motivate for funding from government (National Treasury) and donors (Solidarity Fund).

Epidemiological data were, however, only one of the factors that were used to make decisions. Economic, religious, social and political factors were also taken into account in decision-making. A key lesson was the need to empower decision-makers at the ground level to ensure that interventions were rapidly implemented and context appropriate. While the data systems provided invaluable support, there were challenges and limitations to setting up well-defined systems in time.

Many provinces experienced difficulty with data capturing, highlighting the need for increased personnel. The presence of champions and regular oversight were important for facilitating implementation.

While provincial allocations of cases were complete and robust, allocation errors occurred at more granular spatial resolutions.^[11]^ Improving allocation and mapping systems, as well as co-ordinated central systems of databases of determinants of hospital capacity, such as bed availability, required staff and equipment, will result in resilient and strengthened systems for future health emergencies. Ensuring uniform sustainable systems across provinces, with the associated human and financial resources for implementation/escalation, is important for responding to similar public health threats.

## Conclusion

Rapid development of robust data systems was necessary to support the response to the SA COVID-19 epidemic. These systems produced data streams and supported development of models that were used in decision-making at all levels of government. While much progress was made in producing epidemiological data, many challenges remain and need to be overcome to address gaps and better prepare for future waves of COVID-19 and other health emergencies.

## Figures and Tables

**Fig. 1:**
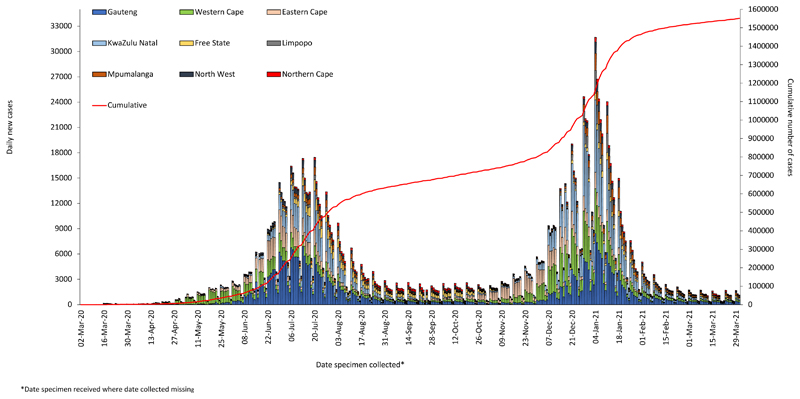
Daily reported COVID-19 cases by province and cumulative national case numbers in South Africa, 2020 - 2021. Source: NMCCS database (NICD).

**Table 1:** Type of report, and frequency of publications using data from national line list and DATCOV database, March 2020 - March 2021

Report	Frequency	Content covered
National COVID-19 daily report	Daily	Changes in number of tests and distribution of cases reported in the past 24 hours, 7-day moving average and cumulative numbers by province
Weekly COVID-19 epidemiologic brief	Weekly	National and provincial trends of COVID-19 cases of all ages in SA by age group, type of health sector and sex
COVID-19 in children surveillance report	Initially bi-weekly. Moved to quarterly in 2021	Epidemiological characteristics of individuals aged ≤19 years with laboratory-confirmed COVID-19 who were notified through the national notification system in SA, and the individuals aged ≤19 years hospitalised for COVID-19-related illness at sentinel hospitals in SA
Weekly COVID-19 testing summary report	Weekly	Weekly COVID-19 testing volumes, positive tests and proportion testing positive by province, district and subdistrict. Report also includes age and sex distributions and laboratory turn-around times
Weekly COVID-19 hospital surveillance (DATCOV) report	Initially weekly publication, moved to bi-weekly towards the end of 2020	Epidemiologic and geographic trends of COVID-19 admissions and in-hospital deaths
The daily COVID-19 effective reproductive number (R) in SA	Approximately monthly	Trends in estimated effective reproductive number by week and province using data on numbers of cases, hospitalisations and deaths
SACMC Epidemic Explorer national and provincial reports	Three times a week	Analysis of resurgence risk, presenting metrics to prepare for future outbreaks, and monitoring COVID-19 hospital admissions and deaths at a provincial, district and subdistrict level
